# Report on Surgery

**Published:** 1874-08

**Authors:** J. L. Hays

**Affiliations:** Paris, Ill.


ARTICLE IV.—*Report on Surgery.* By J. L. HAYS, M.D., of Paris, Ill., May 1, 1874.

*Mr. President and Members of the Æsculapian Society:*

I have the pleasure, as Chairman on Surgery, to report a few cases of some importance.

My first, is one of organic stricture of the urethra in a married male, aged 38. He first found himself with stricture during the war, and was treated by the surgeon of his regiment and cured; near the close of the war he had another attack, when it became so difficult to micturate as to require treatment by catheter; he procured himself a catheter and passed it occasionally until about two months before I saw him in September of last year. He had been informed by some physician that the only way he could be cured was to be operated upon by cutting out the stricture. The physician who had him under charge at the time I saw him, told me he would not consent to have a catheter or sound used—that he was determined to have the stricture cut out with a knife.

I saw him the 19th of September, with a haggard, anxious expression, terrible pain in region of kidneys, bladder largely distended, pulse 100, groaning at almost every breath, and when he walked about the room he went bent over with his hands on the hips. His drawers and bed clothing were wet, and a peculiar odor issued from his person. By standing over a vessel a long while and occasionally straining, enough urine escaped to discover its condition, it being excessively turbid, with muco-pus suspended in it.

I was denied the privilege of sounding the bladder, as he declared no man should touch him but to operate. His treatment previous to this had been judicious, having in view to relieve any congestion or spasmodic condition that might be present. The night previous he had taken a large saline cathartic, and it was followed by an opiate.

When I assured him I would operate, he consented to let me pass the sound. I found a stricture one inch from the meatus, but passing the sound down it stopped in the membranous portion. (The membranous portion of the urethra is three-fourths of an inch long, and lies between the prostate and the bulb.) I manipulated probably half an hour with different sized sounds, but made no entrance. We then put him under chloroform and the sound again stopped at the place as before; I cut down in the raphe to the point of the sound and passed the knife up to the prostate, when the sound slipped in; I then drew off the water and cleaned the catheter and left it in the bladder. Forty-eight hours afterwards I again attempted to pass the catheter, but it would not enter the bladder. We put him under chloroform again and cut a small bridle of the stricture, and passed my finger into the membranous end of the prostate, when the sound (and then afterwards the catheter) passed in readily. I visited the patient the following day and introduced the catheter easily. The next day I failed to introduce the instrument, but as he passed a tolerably good stream through the wound I was satisfied to let him remain.

Although we kept him upon tonics and as good diet as could be had, his health failed, and on the sixth day I thought he would not recover; his condition became very critical, with an anxious, haggard expression, delirous, and pulse feeble at 120. Constitutional treatment was all that could be done; this we kept up, while the flow of urine gradually improved, but most of it passed through the wound. I procured a Holt's dilator, but had the same success—failed to pass it. In four weeks the wound had healed, except a fistulous opening; I closed this by paring the edges, and a suture; in seven weeks after I operated he went to work, being able to pass a moderately large stream, the patient introducing a catheter daily, to the prostate. In February of this year he left the country, and I lost sight of him.

On the 29th of June, 1873, I was called to see a child æt. 6. who had drawn a grain of parched coffee into her windpipe. It had been done three hours before I saw her with Dr. Baum. It was dark when we got to the house. She had been sleeping, but awoke as we entered, and I thought would suffocate, but the paroxysm of coughing having passed, she again fell asleep, and we decided not to operate till morning.

Next morning, Dr. Ten Brook and myself repaired to the place, found the little patient sitting on her mother's lap breathing badly, had had several spells of coughing, but without the effect of expectorating the foreign body. We at once decided to operate, and with the parent's consent, chloroform was administered. We made the operation of tracheotomy. The hemorrhage was considerable although I divided the structures carefully with handle of scalpel; when we reached the trachea, the child had a violent paroxysmal cough and might have suffocated, as it had turned blue and was in a terrible spasm, when I pushed the knife into the trachea, the air whistled in sharply and the child became quiet; cutting about three rings of cartilage and pulling the sides asunder by threads passed through each side, we cleaned out the blood and mucus and found the grain of coffee lodged in the vocal chords of the larynx; after extracting, we closed the wound with adhesive straps, and she made a speedy recovery.

John B., of Indiana, German descent, aged 65, came to my office in the spring of 1872, to ask my opinion in regard to his eyes. I saw he was blind, of cataract, but asked him to give me the history of the case. He said that two years before, he became blind in the left eye and soon noticed the other failing him, which condition continued till he became perfectly blind in a few months afterwards. Some man at Indianapolis, who had some reputation, had gone out to his home and operated on the left eye, but instead of it curing him had made him worse, as he was unable afterwards to see light, and ever since he had suffered with neuralgia and rheumatism.

Upon examining his eyes I found a mature cataract of right eye, pupil perfectly dilatable. The left eye was suffused in tears; pupil nearly closed, with peculiar lustre; iris colored; pupil not affected by atropia; tension increased; conjunctival and sub-conjunctival vessels engorged. and pain in the eye ball and temple. There was no mark of an operation for cataract visible. My diagnosis was easily made, which future developments proved to be true. I had here a case of chronic irido-choroiditis supervening on the operation previously made, and what was the operation? Evidently the operation for cataract by the reclination method.

The question I now had to decide was, is an operation for cataract safe and judicious? This I answered negatively, for the following reason: It has been a well authenticated fact for several years, that a foreign body in the humors of the eye is most likely to produce irido-choroiditis in that eye, and sympathetic ophthalmia in the other, and the hope in such a case is in the removal of the affected eye. To have operated on an eye for cataract in which the other was already blind from irido-choroiditis would have been to sacrifice what little hope was left, and doomed my patient the remainder of his life to darkness.

I therefore advised enucleation of the left eye and a subsequent operation for cataract on the right. He did not consent to this, and returned home. I lost sight of him for about a year, when he returned, still suffering from irido-choroiditis. I again insisted on his having the eye removed, when he reluctantly consented. On May 8, 1873, it was removed, the excessive pain from which he had suffered for so many months left him, and he entirely recovered in about two weeks. On the 30th of the same month, by the assistance of Drs. Miller, Wooley and Baum, I made Liebreich's modified linear flap. The capsule was ruptured, and the lens came out by pressure. I had no prolapse of the iris, and did not make an iridectomy, which is usually done. In five days my patient could bear considerable light. A very slight attack of iritis having occurred in two weeks, I suited glasses to him: for reading, No. 2; for walking and distance, No. 4. The pupil was round, with the exception of a small bit scarcely discernible.

I attribute my success in this case to two things: First, in not being baffled by the patient when he declined to be operated upon rather than have the eye removed; and second, by having full control of the patient when I did operate.

My next case, is E. M., blacksmith, who, six years before I saw him, had a piece of steel or iron scale pass into the eye while at work on the anvil. A physician was summoned, who put him in a dark room and treated him perhaps judiciously; the result, however, was, he went blind from iritis; the attacks of iritis would recur on account of posterior synechia and occlusion of the pupil. The choroid in course of time became involved; this produced or resulted in the same sort of case as before stated, irido-choroiditis. He kept his bed two months; when I saw him I advised an iridectomy, as promising some relief; this was followed by so much relief that he went to work, but it again became as bad as before. I then made a large iridectomy in the upper portion of the iris, which promised even better results; but eventually, after he had been at work two weeks, the pain recurred, and he sent for me to take the ball out; this was done early last spring. A few months later, I procured for him an artificial eye which serves him a good purpose, and I am pleased to say he is satisfied.

